# Engineering Resilient Community Pharmacies for Chronic Care Management: Protocol for the Development of a Medication Safety Map

**DOI:** 10.2196/69011

**Published:** 2025-09-11

**Authors:** Michelle A Chui, Maria E Berbakov, Aaron M Gilson, Jamie A Stone, Elin C Lehnbom, Emily L Hoffins, Katherine G Moore, James H Ford II

**Affiliations:** 1 Chui SAMS (Systems Approach to Medication Safety) Lab School of Nursing University of Wisconsin–Madison Madison, WI United States; 2 Sonderegger Research Center School of Pharmacy University of Wisconsin–Madison Madison, WI United States; 3 Division of Social and Administrative Sciences School of Pharmacy University of Wisconsin–Madison Madison, WI United States; 4 Department of Pharmacy Faculty of Health Sciences UiT The Arctic University of Norway Tromsø Norway; 5 Department of Pharmacy Faculty of Pharmacy Uppsala University Uppsala Sweden

**Keywords:** medication safety, community pharmacies, provider status, pharmacy intervention, participatory design, stakeholder groups, resilience, safety-I and safety-II

## Abstract

**Background:**

The increase in people with complex chronic health conditions is stressing the US health care delivery system. Community pharmacies play a role in ensuring patients’ safe medication use for chronic care management (CCM), but their efforts are undermined by volatile work demands and other system barriers. Medication safety in community pharmacies is a multidimensional issue shaped by the work system and interactions among pharmacists, primary care providers, and patients.

**Objective:**

The objective is to create and evaluate a system of CCM that supports safe medication use. The CCM system design will focus on creating and evaluating a Medication Safety Map (MedSafeMap) for patients with complex chronic health conditions. This study has three aims: (1) identify and define community pharmacy work system design requirements for safe medication practices, enabling resilient performance; (2) design and develop MedSafeMap, a feasible and sustainable solution, to facilitate safe medication practices through resilient performance; and (3) implement MedSafeMap in community pharmacies and pilot-test its impact on pharmacy staff attitudes, behaviors, and performance.

**Methods:**

This study will leverage participatory design and human factors engineering methods throughout the 3 aims. For aim 1, four rounds of qualitative observations within 6 pharmacy sites will be conducted to parse areas MedSafeMap could address. Two rounds of interviews with pharmacists and technicians from each of the sites will be used to expand upon areas of interest identified during the observations. Observational and interview data will be used to construct functional resonance analysis method models and resilience narratives to map both risks and best practices within the system based on daily workplace factors. For aim 2, focus groups with pharmacist and technician stakeholders will be guided by participatory stakeholder engagement to inform prototyping for MedSafeMap. Simulation-based research involving standardized patients in CCM scenarios will be used to test and refine MedSafeMap components. Finally, for aim 3, MedSafeMap will be implemented in pharmacies. Observations using the work observation method by activity timing (WOMBAT) for the time and motion study will aid in understanding how MedSafeMap impacts pharmacy staff workflow. We will assess adoption challenges and resilience-focused attitudes, behaviors, and performance to support CCM.

**Results:**

As of August 2025, all 6 pharmacy sites have been recruited. Three of the 4 rounds of observations, 2 rounds of interviews with 12 pharmacists and 12 technicians from the study sites, and the 6 focus groups have been conducted. Preparations for the simulations are ongoing.

**Conclusions:**

MedSafeMap is an innovative approach that will guide pharmacists and technicians in safely providing care to patients with complex chronic health conditions. It will help them navigate the complex tasks and communications between the pharmacy, patient, and primary care provider arising with this type of complex care.

**International Registered Report Identifier (IRRID):**

DERR1-10.2196/69011

## Introduction

### Background

More than 100 million Americans have multiple chronic conditions [1] that contribute to more than 1 million adverse drug events (ADEs) annually, prompting 4 million people to seek medical care, costing the US health care system >US $8 billion per year [2,3]. Health care spending for patients with multiple chronic conditions is up to 20 times higher than for patients with no chronic conditions; that is, Americans with 5 or more chronic conditions comprise 12% of the population but contribute to 41% of the total health care spending, averaging 20 physician visits per year and using up to 50 times more prescription medications [1]. Challenges with managing multiple chronic conditions have a direct impact on health care use, with such patients representing a quarter of all hospital admissions annually, with one-third of all patients having at least 1 emergency department visit every year [1]. Despite these health consequences, efforts to improve medication safety for patients with complex chronic health conditions in outpatient settings have not been allocated appropriate resources to be effective.

Community pharmacists are recognized as the champions of medication safety not only for performing dispensing tasks but also for their expanding role in chronic care management (CCM) [4]. CCM can include managing health problems and goals, medications, other health care providers, and community services that patients have and need. In many states, CCM also encompasses ordering and interpreting tests for chronic conditions, such as diabetes [4], through collaborative practice agreements with health care providers and ensuring patients receive appropriate screenings and immunizations. Such expanded practice was facilitated by the COVID-19 pandemic due to pharmacists being one of the few health care professionals providing face-to-face care and assuming clinical responsibilities when primary care physicians and nurses were managing COVID-19 emergencies [5].

Furthermore, community pharmacists play a vital role in medically underserved communities [6], such as in locations with too few primary care providers, high poverty, or a high older adult population. Patients in these areas face difficulties or delays in getting basic health care because of long travel distances to health care providers, long wait times for appointments, or a lack of health care providers who can serve uninsured or underinsured patients. Therefore, pharmacists are considered accessible primary care practitioners, seeing patients 5 to 8 times more frequently than primary care physicians [7]. Numerous studies have demonstrated the ability of pharmacists to address medication nonadherence [8], support naloxone provision and opioid safety counseling [9], provide pain management and palliative care [10], improve diabetes and cardiovascular outcomes [11], and improve vaccination rates in medically underserved communities [12,13].

However, the abilities of community pharmacists to fill this gap are constrained by a work system that is unsustainable and essentially broken [14]. Pharmacies adhere to product-based reimbursement contracts with pharmacy benefit managers, incentivizing pharmacy staff to fill more prescriptions. Therefore, for more than a decade, pharmacists have reported high workload and burnout, with opaque corporate performance metrics based on prescription volume and customer service [14] rather than on safety or clinical outcomes. The COVID-19 pandemic exacerbated pharmacists’ workload strain by adding testing and vaccinations to a system that is chronically understaffed and stretched beyond capacity. With pharmacist burnout rates at an all-time high, a call for action to improve pharmacist work systems is critical to prevent a complete collapse in the US pharmacy system. Pharmacists across the United States have been executing a series of walkouts since September 2023 to improve harsh working conditions and preventable errors in care [15]. In Walgreens alone, 600 employees across 20 stores have participated in walkouts, resulting in multiday disruptions in workflow and patient care [15]. The purpose of these walkouts is to shed light on the need for a solution to improve the working conditions of pharmacy staff.

In light of pharmacists’ unmanaged workload, there is a push for them to do more. This is evidenced by the introduction and recognition that pharmacists are health care providers (eg “provider status”) [16]. This status will allow pharmacists who provide services such as CCM, comprehensive medication management, and immunizations to receive reimbursement for such clinical services in addition to product-based dispensing services [17]. This provides a timely opportunity for pharmacists to envision innovative designs to improve medication safety for their patients. To reimagine community pharmacies as an accessible destination for CCM, we must recognize the increased complexity of the community pharmacy work system. Similar to the increase in pharmacy staff responsibility related to the COVID-19 pandemic response, adding more clinical services with the addition of reimbursement has potential implications for the quality of pharmacist education, business practices, and safe medication dispensing. Opening up avenues for reimbursement that are not product based allows stronger sustainability of expanded services and greater impact in their patient care practices, which can potentially fill a critical gap in Wisconsin, where two-thirds of its counties are medically underserved [12,13].

This opportunity to adopt new services and create coordinated care models can present a significant medication safety challenge. Community pharmacists’ heroic response to the COVID-19 pandemic demands created work systems that increased stress and negatively impacted their ability to ensure safe use of medications they dispense [18]. Pharmacist provider status can lead to sustainable and collaborative programs that holistically address chronic care conditions of patients with complex chronic health conditions. However, doing so without prospectively considering and testing unintended consequences may negatively transform the community pharmacy work system, threatening the very medication safety strategies on which pharmacists have come to rely. The consequence of this reality is synergistic; high workload demands contribute to medication errors and ADEs, leading to pharmacists experiencing “learned helplessness” and a lack of resiliency (or the ability to adapt to unpredictable work situations) in the face of these ADEs [19-22].

### Approaches to Medication Safety

Many medication safety studies strive for “zero harm” [23-25] by focusing on “what went wrong” in the face of undesired outcomes, referred to as a “safety-I” approach [26]. The traditional harm-reduction approach has been to conduct root cause analyses of errors and understand the contributing factors that led to the error to reduce their incidence or mitigate their harm. A number of strategies have been encouraged based on this approach, including those developed and tested in our previous studies [27,28], but in the end, these frequently result in more constraints in a complex system that limit workers’ ability to adapt. Such strategies end up limiting the capacity to adapt to practice demands and make the system more brittle, which can unintentionally lead to other medication safety hazards. Furthermore, stacking isolated interventions does not lead to fundamental changes to the approach to medication safety. It is not surprising that little progress on substantive improvement has been made in medication safety in the outpatient community pharmacy setting [29].

Medication safety must be considered in a new way that encompasses safety-II, the “what went right approach.” Safety-II [30] is based on resilience engineering, which focuses on understanding how resilient performance is achieved, creating a work system that will address unexpected threats while also responding to opportunities [31]. Instead of framing safety as a linear, failure-based model, we need to broaden the scope of inquiry to recognize the complexity and inherent variability in modern systems, such as community pharmacies. Furthermore, we need to shift beyond an exclusive consideration of ADEs and failures to understand and strengthen a work system’s abilities to continuously create safety in everyday practice [32]. This goal is achieved by focusing on the abilities of a work system to absorb significant stress before a disruption impacts safety, return to normal operations as quickly as possible, and, importantly, adapt to undesirable situations. A system with adaptive capacity can anticipate disruptions and reorganize workflows using alternative paths to maintain successful work and minimize disruptions [33]. An example of safety-I and safety-II approaches can be found in Textbox 1.

**Examples of safety-I and safety-II approaches.**

**Safety-I example**

- Checklists can decrease risk by imposing a structured process but may be counterproductive
- A checklist can be completed by rote rather than thoughtfully
- A checklist may instill a false sense of security when there is an assumption that all important items are contained in the checklist
- Current conditions may be different from the conditions under which the checklist was conceived
- The ability for workers to adapt to new conditions is lost

**Safety-II example**

- A checklist restricts an unexpected situation
- Workers double-check facts against each other
- Workers are open and honest about explaining why they wish to deviate from the checklist
- There is a healthy skepticism about deviating from the checklist
- Workers ask for constructive criticism, ensuring that they are taking the best possible novel approach
- Workers embrace team members from other work systems for their outside the box thinking

### The Patient Safety Learning Laboratory: Engineering Resilient Community Pharmacies

Despite numerous calls, there has not been a systematic engineering approach toward outpatient medication safety within community pharmacies.

Previous patient safety learning laboratories (PSLLs) have evaluated the physical environment, sociotechnological factors, and clinical workflow within inpatient and clinical settings but not within the outpatient community pharmacy settings [34]. This project, informed by resilience engineering, will address the challenges common in community pharmacies to achieve safe medication use in community-dwelling patients with multiple complex chronic conditions. The goal is to evolve from the safety-I approach to a combined safety-I and safety-II approach. The resulting outcome, the Engineering Resilient Community Pharmacies (ENRICH) PSLL, is expected to gain knowledge of strategies for building resilience capacity in community pharmacy work systems in conducting CCM. PSLLs encompass a systematic engineering approach to allow cross-disciplinary professionals to evaluate clinical processes and approaches toward improvements in patient safety [34].

Our long-term goal is to position the ENRICH PSLL as the premier center for medication and patient safety and CCM in community pharmacies. Despite our previous work, a current gap exists in the systematic application and evaluation of human factors and health systems engineering approaches to improve medication safety and staff work life in community pharmacies. Our PSLL, which is led by experts in human factors and health systems engineering, represents a foundational step toward the creation of a center to promote and normalize a safety-I and safety-II approach in pharmacy systems research. Our PSLL will include a focus on the use of the principles of dissemination and implementation research to enhance the potential for sustainability of improvements resulting from patient safety innovations. In addition, this PSLL is designed to generate ideas for future research projects and examine the implications and outcomes related to pharmacists’ provider status, which can serve to expand CCM to support patients with complex chronic health conditions living in medically underserved areas. The PSLL resulting from this study represents the beginning of a new paradigm, unprecedented in the pharmacy setting, for improving medication safety for community-dwelling patients with complex chronic diseases.

## Methods

### Design Overview

**Figure 1:**
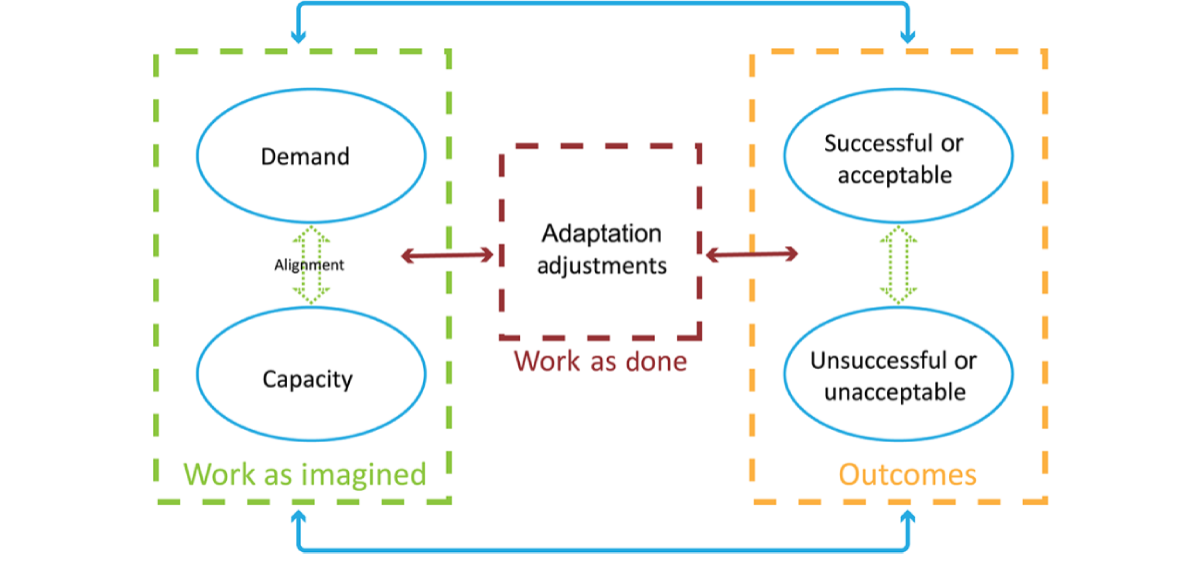
Concepts for Applying Resilience Engineering model.

The methods used in this project have demonstrated success with other health care practitioners and in other patient safety studies [27,28]. The concepts for applying resilience engineering (CARE) model guides our data collection, analysis, and interpretation (Figure 1). Resilience is the ability of the community pharmacy work system to adjust its functioning before, during, and following sentinel or unexpected events and thereby sustain operations under both expected and unexpected conditions [35]. It assumes that variability in the environment creates the need for adjustment. Work as imagined (WAI) is conceptualized as the intended or imagined alignment between demands of the system (prescription volume and patient acuity) and the capacity to meet those demands. Demand and capacity can never completely align because of the complex nature of the system; there will always be unforeseen demands that require workers to adjust in situ. In addition, workers do not simply comply with protocol but naturally adapt and innovate as part of taking control over their environment. In the CARE model, work as done (WAD) refers to the adjustments and natural variability in how tasks are conducted. Predicting acceptable (what goes right) and unacceptable (what goes wrong) outcomes depends on an understanding of WAD in different demand and capacity circumstances. In this project, we will be using the concepts of WAI and WAD to identify the gap in how pharmacy staff plan their approaches to patient care and how they are able to actually fulfill their responsibilities (Figure 1).

**Figure 2:**
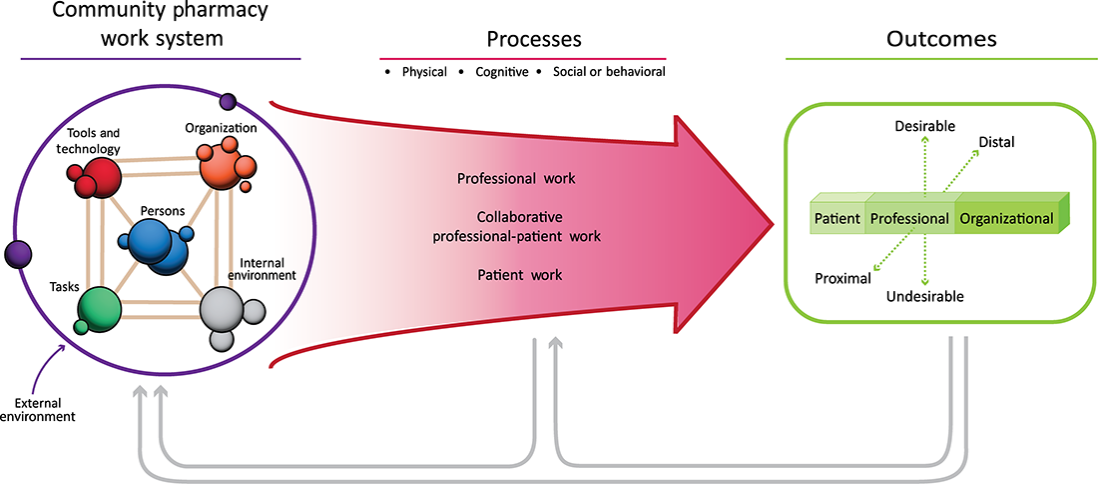
Systems Engineering Initiative for Patient Safety 2.0 model adapted for the community pharmacy work system.

Because resilience also applies to the organizational level and adaptations in complex systems, it is essential to ensure that characteristics and adaptations of the entire work system are captured. To operationalize the discrete facets of the work system, we have used systems engineering initiative for patient safety (SEIPS) 2.0, a human-factors engineering model to improve patient outcomes (Figure 2 [36]). The SEIPS model has framed the design and analysis of many safety studies, including the research we conducted in community pharmacies [28,37-39]. The CARE and SEIPS models work well in tandem because both models contain feedback loops and account for the interconnectedness of work system components. In Figure 2, *professional work* references both pharmacists and technicians. Patient work includes the work of the patient and their caregiver, which is essential to CCM (Figure 2).

### Ethical Considerations

Ethics approval for human participant research will be obtained from the University of Wisconsin–Madison Institutional Review Board (S-2023-1100, S-2024-1747). Each aim of this study will be individually submitted to the institutional review board (IRB) for review and approval before initiating the research activities concerning each aim.

#### Human Participant Ethics Review Approvals

##### Aim 1: Observations in the Community Pharmacies, Pharmacist and Pharmacy Technician Interviews, and Pharmacy Staff Demographic Sheets

Aim 1 received ethics approval from the University of Wisconsin–Madison Institutional Review Board (S-2023-1100) on August 7, 2023. Research activities for this aim were conducted in accordance with the University of Wisconsin–Madison ethical regulations. Several days before observation, the study’s primary contact at each community pharmacy was provided with a sample email script for notifying pharmacy staff about the purpose of the observations and when they would be taking place. Upon arrival at the pharmacy, researchers introduced themselves to the staff, explained the purpose of the observation, and clarified observation duration and how staff anonymity is preserved. Pharmacist and pharmacy technician interviews were conducted via Zoom (Zoom Communications, Inc) at a time most convenient for the participant. Participants were provided with an IRB-approved information sheet and waiver of signed consent detailing the study’s purpose, tasks required for participation, and information regarding confidentiality protection. They were also informed that their participation was entirely voluntary, and they all had the right to withdraw from the project at any time without any negative consequences. Before the interview, they were also provided with an interview guide for either the first (Multimedia Appendix 1) or second (Multimedia Appendix 2) round of interviews.

##### Aim 2: Pharmacist and Pharmacy Technician Participation in Stakeholder Focus Groups, Pharmacy Staff Demographic Sheets, and Simulations

Aim 2 received ethics approval from the University of Wisconsin–Madison Institutional Review Board (S-2024-1747) on January 2, 2025. Research activities for this aim are being conducted in accordance with the University of Wisconsin–Madison ethical regulations. Participants are being provided with an IRB-approved information sheet and waiver of signed consent detailing the study purpose, tasks required for participation, and information regarding confidentiality protection. In addition, participants are being informed that their participation is entirely voluntary, and they all have the right to withdraw from the project at any time without any negative consequences.

##### Materials for Aim 3

Materials for aim 3—Medication Safety Map (MedSafeMap)’s implementation into pharmacies and pre- and postimplementation observations using work observation method by activity timing (WOMBAT) for time and motion study and postimplementation evaluations—are still in development and will be submitted for ethics approval from the University of Wisconsin–Madison Institutional Review Board before the start of research activities concerning aim 3. The anticipated timeline for submission of aim 3 study materials to the IRB is December 2025 and will include a script for observers to read to pharmacy staff, detailing a brief introduction, the purpose of the observation, the duration of the observation, and how staff anonymity will be preserved.

#### Privacy and Confidentiality

Throughout all project research activities, all participant information will be anonymized with the use of participant numbers and in accordance with the university’s policies. Additional protection measures include secure storage of data per the university’s policies, limiting data access to only approved study personnel and ensuring there is no participant-identifiable information included in publications.

We will adhere to the National Institutes of Health Grants Policy Statement on *Availability of Research Results: Publications, Intellectual Property Rights, and Sharing Resources*. We will take into account any additional policies and rules including but not limited to institutional policies; local IRB rules; and local, state, and federal laws and regulations. We acknowledge that the rights and privacy of people who participate in National Institutes of Health–sponsored research must always be protected. Thus, data intended for broader use will be free of identifiers that would permit linkages to individual research participants and variables that could lead to deductive disclosure of the identity of individual participants.

#### Participant Compensation

Each pharmacist and pharmacy technician who participated in an interview was paid US $50 via an electronic gift card. For the focus groups, pharmacists and pharmacy technicians were or will be paid US $80 in cash for the first, second, third, and fourth meetings. They will each be paid US $120 in cash for the fifth meeting and US $160 each for the final meeting. For the simulations, each pharmacist and pharmacy technician will have the opportunity to participate in up to 3 simulation sessions, earning US $150 each for their participation in each session. For the post implementation interviews during aim 3, pharmacists and pharmacy technicians will each be paid US $50 via an electronic gift card. Details on compensation are included in both recruitment communications with the participation and the information sheet that is provided to the participant upon requesting more information on the study.

### Study Sites

To ensure a complete understanding of the problems and implementation strategies of varied community pharmacy work systems, we will partner with 3 pharmacy organizations. First, Advocate Health is the 10th largest not-for-profit, integrated health system in the United States, with 70 community pharmacies that are geographically dispersed throughout urban and rural Wisconsin and Illinois. Second, UW Health is the integrated health system of the University of Wisconsin–Madison, with 12 outpatient pharmacies. Third, Boscobel Pharmacy and Center Pharmacy are located in rural southwest Wisconsin and are independently owned.

Within each of these organizations, 2 pharmacies will participate that serve ethnically diverse populations and people in medically underserved areas that have high area deprivation indices based on income, education, employment, and housing quality for 6 pharmacy sites. In addition, all pharmacies will have at least read-only access to the electronic health records of their patients, which may serve as a tool to facilitate CCM.

We will also partner with Fitchburg Family Pharmacy to conduct in situ simulations, in which we will use simulated scenarios of patients with complex chronic health conditions in a pharmacy itself rather than in training facilities for the design and development phases (aim 2) of the project. Fitchburg Family Pharmacy is a medium-sized urban independent pharmacy that provides CCM to a diverse patient population.

### Project Aims

Table 1 describes our project structure, starting with problem analysis (aim 1) and continuing with design and development (aim 2), which will lead to implementation and evaluation (aim 3). An expert advisory board, comprising transdisciplinary stakeholders, will be formed to provide us with high-level consultation and content expertise for each of the 3 project aims. This board includes patients, caregivers, a pharmacy technician researcher, a pharmacy owner, primary care physicians, managers of independent, local, and chain pharmacies, and leaders of pharmacy and medical societies in Wisconsin.

**Table 1:** Project structure guided by the Agency for Healthcare Research and Quality Request for Application 5-step methodology.

	Problem analysis (step 1)	Design (step 2) and development (step 3)	Implementation (step 4) and evaluation (step 5)
Aims	Identify and prioritize (aim 1)	Prototype and simulate (aim 2)	Pilot and evaluate (aim 3)
Methods	Observations and interviews Information flow diagraming Artifact analysis	Participatory design Simulation	Time motion Before and after evaluation Expert evaluation Surveys and interviews
Outputs	Resilience narratives Functional resilience analysis method diagrams	Prototypes Ideal interactions Detailed design specifications	Impact of prototypes on process and outcomes Tools to assess capacity for resilience Implementation guide

### Aim 1: Identify and Define Design Requirements

#### Overview

To develop effective and sustainable interventions, an in-depth problem analysis of the work system must be conducted. Thus, aim 1 seeks to understand and map work system resilience, including how WAI differs from WAD, how adjustments are created, and how outcomes are generated. Iterative data collection and analysis will allow continual refinement and clarification of the work system.

#### Observations

In-depth ethnographic observations will be conducted over 4 visits to each of the 6 pharmacy observation sites (Textbox 2). Study team members will debrief after each observation as well as in situ as needed to adjust observation approaches or focus on specific aspects of the workflow. We will complete 24 hours of observations over 4 days. Observed artifacts that reflect WAI, such as policies and procedures, memory aids, and cognitive tools, will also be collected and recorded. Full descriptive field notes will be produced immediately after each observational session. Data will be collected concurrently at each setting, and researchers will work across all settings so that comparisons between the settings can inform ongoing data collection strategies.

**Observations at the pharmacy sites.**

**High-level observation questions**

- What contributes to variability in demand and capacity?
- What misalignment occurs between demand and capacity and why?
- What pressures, problems, or goals are pharmacists and technicians responding to when they create new ways to achieve outcomes?
- How and under what circumstances do adjustments or adaptations lead to successful and unsuccessful outcomes?

**Visit 1: exploratory evaluation**

- Observe the pharmacy environment holistically
- Identify staff roles and responsibilities, processes, procedures, flows of information and communication, coordinating mechanisms, and supporting tools and technology
- Identify interest areas for more precise and targeted observations in subsequent stages

**Visits 2 to 4: observations**

1. Observe the pharmacy staff
2. In-depth observations of important processes, such as shift-change handoffs and medication reconciliation for patients during hospital discharge, as processes critical to providing chronic care management
3. Short, structured discussions and facilitated reflection from staff on aspects of the concepts for applying resilience engineering model and using the systems engineering initiative for patient safety model components

#### Interviews

Semistructured interviews will be separately conducted with 2 pharmacists and 2 technicians from each of the 6 pharmacy locations to explore and follow up on issues from the previous observation stages to identify processes or areas for additional observation. Questions for the first round of interviews will focus on how people do their work and how they manage tricky situations, with probes about variability, adaptations, problems, and challenges (Multimedia Appendix 1). Questions for the second round will focus on staff experiences with, understanding of, and expectations for specific nondispensing services within community pharmacies (Multimedia Appendix 2). Interviews will be audio recorded and transcribed for analysis with participants’ consent.

#### Data Analysis

Data analysis will proceed in 3 stages. First, a combined deductive-inductive approach will be used to thematically analyze the observation and interview data. A coding scheme will be developed based on elements of the SEIPS model as well as important themes in the data not captured by the model (Table 2). Categories will be developed by constantly refining the coding scheme, and comparisons will be made between different respondents, different pharmacies, and different processes.

**Table 2:** Aim 1 observation and interview coding scheme.

Levels	Comparison of WAD^a^ and WAI^b^
Teams of pharmacists and technicians	Interpersonal communication, including verbal and nonverbal Teamwork skills, including leadership and situation awareness
Technology	Usability, flexibility, adjustability, and adaptability
Internal environment	Interruptions and distractions Lighting Physical ergonomics of the workspace Crowding and disorganization
Tasks	Task demands and workload Contextual changes, including situation deviations from normal or expected Production pressure
Organization	Complexity, uncertainty, and risk Information flow, including quantity and pacing Culture and climate Policies and procedures Incentives and disincentives

^a^WAD: work as done.

^b^WAI: work as imagined.

Second, guided by the CARE model, we will expand on the SEIPS coding scheme to construct functional resilience analysis method (FRAM) models [36,40] to visualize WAD and articulate the discrepancies between WAI and WAD. FRAM models allow all activities within selected processes to be visualized to show how they relate to each other and how they interact, based on six aspects, namely (1) input, (2) output, (3) precondition, (4) resources, (5) time, and (6) control, demonstrating the system’s complexity [41]. Each process within the community pharmacy is linked, which allows the study of the variability of each process. That variability can produce amplified and unpredictable outcomes (either acceptable or unacceptable) [36,40].

Third, the themes generated by the deductive-inductive approach and the FRAM models will be used to create resilience narratives describing trajectories of activity linking misalignments of demand and capacity, adjustments, adaptations, and outcomes. The narratives will focus on how adjustments and adaptations (WAD) mediate between pressures caused by misalignments of demand and capacity and outcomes.

### Aim 2: Design and Develop MedSafeMap

#### Overview

Stakeholder-engaged participatory design and simulation-based research will be used in an iterative fashion to design and develop MedSafeMap, a package of components to optimize pharmacy staff interactions within their work system and with patients, and detailed pharmacy design recommendations. Standardized scenarios of patients with complex chronic health conditions will be developed to have both internal validity and fidelity to the real-world pharmacy environment. The goal is to create MedSafeMap components that will ultimately lead to pharmacy staff resilience and optimized patient interactions around CCM.

#### Participatory Design

Participatory design is an approach to system design that actively involves all stakeholders at several stages of the innovation process to help ensure that the result meets their needs and is usable [28]. Two stakeholder groups will be used: one group consisting of 5 community pharmacists and another group consisting of 5 technicians.

Stakeholder group meetings will be held 6 times, approximately every 6 to 8 weeks. The first 4 meetings will be held with the pharmacists and technicians separately because they have different work responsibilities, tasks, and concerns. We also want technicians to fully engage in MedSafeMap development without a pharmacist’s presence imposing a power differential. The resilience narratives and FRAM diagrams developed in aim 1, which demonstrate variability and resilience potential, will inform the design and development of the MedSafeMap. We will focus on the processes required to conduct CCM for patients with complex chronic health conditions, including the possibility of a communication tool to support a discussion of alternatives, enhanced instructions for patients, and cognitive aids to support memory. The final 2 meetings will involve MedSafeMap refinement. To do so, we will bring the pharmacist and technician groups together to work collaboratively. Textbox 3 provides a list of anticipated or possible MedSafeMap components. Completed MedSafeMap components will be peer reviewed by our advisory board to confirm that they will reproduce the experience in a real-world pharmacy. Scenarios will be piloted in the in situ simulated setting and refined before implementation in aim 3.

**Current interactions and potential Medication Safety Map (MedSafeMap) components to encourage ideal interactions.**

**Current interactions**

- Chronic care management seen as important but has an impact on workload, which is a barrier to sustained services.
- Fragmented information across health care providers
- Medication information gap (eg, multiple pharmacies, no diagnoses, or laboratory values)
- Busy retail pharmacies lead to reduced interactions with patients
- Coordination gap between pharmacists and caregivers, patients, and health care providers
- Technicians limited by traditional roles
- Medication concerns not adequately addressed
- Limited support and space for shared decision-making

**Potential MedSafeMap components**

- Modeling behaviors: tactics to overcome communication barriers in pharmacies and engage health care providers; patient and family support to bridge the information gap
- Information support: use technology or low-technology strategies to push eligible patients into the pharmacy workflow; create mechanisms that provide caregivers the opportunity to be involved before, during, and after an appointment
- Cognitive aids: physical structures to improve pharmacists’ and technicians’ situation awareness; supports in addressing high-priority medication needs
- Collaborative work support: facilitate a huddle between pharmacist and technician to create an appointment agenda, anticipate patient challenges, and review monitoring needs
- Role clarity and cross-training: empowering technicians as equal partners and leaders in the community pharmacy

#### Simulation-Based Research

In situ simulation [42], or simulation in an actual community pharmacy, will then be conducted to study the ergonomics and physicality of the work setting on performance. In situ simulations will also promote process and MedSafeMap refinement before deployment in actual pharmacies.

**Figure 3:**
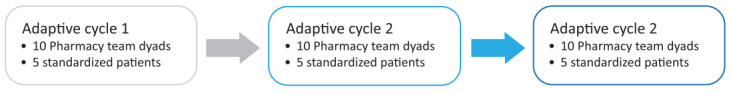
Simulation cycles.

The final 2 stakeholder meetings will involve 3 rapid adaptive simulation cycles for MedSafeMap refinement (Figure 3). Each simulation cycle will include 5 different standardized patients (hired for the study). Each patient will represent 1 of the 5 different complex CCM scenarios that will include at least 2 chronic conditions and at least 1 social determinant of health (eg, a low health literacy patient with diabetes and congestive heart failure who is nonadherent to medications). Staffing and equipment, level of complexity, and other critical indicators identified in the participatory design will vary and allow for comparisons across the 5 patient scenarios. Creating robust and standard scenarios is important to mitigate threats to internal validity. Thus, we will devote considerable time to ensuring that MedSafeMap may be used in different patient cases and different community pharmacy settings (ie, is pharmacy agnostic; Figure 3).

In total, 10 pharmacist and technician dyads will be recruited to participate in each adaptive simulation cycle. In each adaptive cycle, the pharmacy and technician dyad will complete the simulation 5 times, once for each standardized patient. The process will be replicated for each of the 10 pharmacy dyads, resulting in 50 simulations per adaptive cycle. At the completion of each cycle, the study team will use the data collected, combined with significant reflection and formative evaluation, to refine MedSafeMap that facilitates resilience and encourages adaptation by pharmacy staff.

#### Data Collection

Four wide-angle video cameras will be used to record the simulation from locations that best capture how pharmacy staff interact with the physical space, communicate, share information, and use artifacts within the work system. In addition, 2 sets of eye-tracking glasses (Tobii Pro Glasses) [43-45], one each for the pharmacist and the technician, will be deployed to track eye movement in the natural world, which will inform the development of prototypes. Specifically, as pharmacy staff navigate the physical pharmacy space, the glasses collect data that will provide an understanding about which artifacts are most instrumental to performing CCM, factors that influence pharmacy staff and patient decision-making, and workflow. Gaze data will be recorded as pharmacy staff read and review information on prescriptions and prescription profiles in the pharmacy electronic health record and interact with and educate patients. Aim 2 will be accomplished in 3 steps, conceptualized in Table 3.

**Table 3:** Aim 2 data collection procedures.

	Step 1	Step 2	Step 3
Tasks	Record simulations and document individual and collective behavior	Introduce MedSafeMap^a^ and record simulations: simulate with 10 pharmacist and technician dyads for each of 5 different standardized patients	Conduct individual debrief interviews with the pharmacists and technicians following completion of each simulated scenario
Goal	Identify patterns of interactions and thought processes	Reproduce situations identically to increase confidence in the MedSafeMap’s ability	Gain insights into the thoughts and cognitive processes of pharmacists and technicians
Output	Descriptive account of how community pharmacy staff make decisions or share information; understand rare events, such as sentinel medication errors	Explain variation in the outcome of interest	Understand participants’ perceptions of (1) understanding the problem, (2) assessing prototype feasibility, (3) evaluating the effect of the prototype, and (4) optimizing the design and implementation of the prototype

^a^MedSafeMap: Medication Safety Map.

#### Data Analysis

Observational checklists will be used to code participant behavior recorded on the videos, thereby assessing MedSafeMap’s feasibility and effectiveness. The recordings from the wide-angle video cameras and eye-tracking glasses will be analyzed for themes among participants and across sites by assessing which areas of interest participants fixate on for extended periods. Using gaze duration as a proxy for the wearer’s attention provides insights into which elements of MedSafeMap are influential in shaping pharmacy staff’s behavior. For example, if one of the components of MedSafeMap includes a technician training to speak up when they are concerned that the pharmacist missed a medication-related problem, we can respectively code the reactions of the pharmacist and technician based on the videos [46,47].

Qualitative data from pharmacist and technician stakeholder meetings will be subjected to a rigorous analytic approach [37,48-51]. We will use deductive content analysis, inductive patterns, themes, and categories in our data, guided by our theory-based approach [52]. A conceptual coding structure will be created, and as each interview transcript is added to the analysis, passages will be classified according to existing codes, and codes will be added as needed. Inductive content analysis will also be conducted to determine unanticipated work system characteristics that may facilitate or inhibit resilience.

### Aim 3: Implement and Pilot-Test MedSafeMap

#### Overview

Although simulation-based research can have high internal validity by controlling the highly standardized scenarios that will be developed in aim 2, it is critical to implement and evaluate MedSafeMap in real life, where WAD diverges from WAI, and address external validity concerning pharmacy work systems relevant to CCM. With this approach, we can determine whether changes in performance can be attributed to MedSafeMap rather than other external (eg, regulatory changes) or internal elements (eg, updates to electronic health records) and how such elements can affect MedSafeMap’s performance or impact. The 6 community pharmacies that participated in aim 1 observations and interviews will implement MedSafeMap for optimal interactions designed in aim 2.

#### Implementation and Adaptation

**Figure 4:**
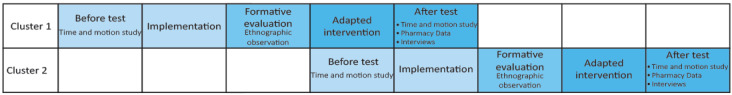
Stepped-wedge design and data collection methods.

A stepped wedge design [39] will be used, where the MedSafeMap will be sequentially implemented in 2 clusters, each cluster comprising 3 pharmacies (1 pharmacy from each organization; Figure 4). The 2 pharmacies from each organization will be randomized into the 2 clusters. Each cluster will commence with baseline pretest data collection and then cross over from the control condition (before implementation) to the intervention condition, where a formative evaluation of MedSafeMap and the strategies from the implementation guide will be conducted. This evaluation will be performed for each cluster 4 months into its implementation period to consider “what might be missing, what sociotechnical factors had not been considered, and to identify ‘bugs and glitches’ that still need to be addressed” [53]. The evaluation will include one day of observations by shadowing pharmacy staff and may involve short discussions and facilitated reflection from staff at each of the 3 pharmacies in cluster 1. Those observations will be reported to the research team and advisory board to identify opportunities to improve MedSafeMap. Following the formative evaluation in cluster 1, we will refine and improve MedSafeMap and implementation strategies to mitigate vulnerabilities or unintended consequences that may expose patients to new harms or further burden the pharmacists and technicians with more work. The adapted MedSafeMap and implementation strategies will be deployed for cluster 2, which will also undergo a similar evaluation. It is possible that minor adaptations will be made, if necessary, before subjecting the cluster to a posttest evaluation [54] (Figure 4).

#### Evaluation

We will use a sequential explanatory design in which the initial data are quantitative (time and motion study and work volume) and complemented by qualitative data (interviews and medication error reporting). This mixed methods approach will allow the research team to understand variables individually and determine which ones will require further analysis. In line with the CARE and SEIPS 2.0 models, we will collect and analyze data that reflect workflow and care processes and the perspectives of pharmacists and technicians.

#### Time and Motion Study

Our evaluation goal is to examine the impact of MedSafeMap on pharmacists’ and technicians’ ability to be resilient, that is, to successfully attend to unexpected threats while also responding to opportunities [31], such as CCM. To do so, we must understand clinical work processes and the way that care is delivered to patients. We anticipate that MedSafeMap will impact the way pharmacists and technicians perform their work and how they engage each other and their patients. Time and motion studies have been used to measure clinical workflow–related factors, including time and task distributions, frequency of multitasking and interruptions, and who completes tasks, with a goal of improving efficiency [55,56].

We will conduct observations before and after the implementation of the adapted MedSafeMap. Using the observational data collected in aim 1, we will develop a pharmacy work task classification (Table 4). The classifications will be incorporated in the WOMBAT program to allow for the consistent recording of observational data [57,58]. WOMBAT allows observers to time the start and end of tasks and record multiple simultaneous tasks using the multitasking function. If an external factor appears to cause a pharmacist or technician to stop performing one task and start another task, an interruption can be recorded.

**Table 4:** Pharmacy work task classifications.

Work task classification	Examples
What: the task being performed	Counseling and communication with the prescriber
Where: the physical location where the task is undertaken	Behind the desk, in the office, and over the counter
With whom: whom the pharmacy staff was with when performing the task	Alone, patient, technician, and student
How: any tools used to complete the task	Phone, face-to-face, and computer

#### Observer Training

Before MedSafeMap’s implementation and baseline data collection, 2 observers will be trained to use the WOMBAT program. Data collection will begin when simultaneous but independent observations of the same events, such as those seen in Table 4, yield an interrater reliability score of at least 85% [59].

#### Data Collection

Observations will be purposely scheduled across a variety of weekday shifts to maximize data variability and capture the full pharmacy workday. One observer will shadow a pharmacist equipped with a tablet with the WOMBAT software installed on it, while, at the same time, a similarly equipped second observer will shadow a technician. In total, 2 pharmacists and 2 technicians will be observed at each pharmacy. Observations will be limited to three 2-hour sessions per day, each separated by a brief break to reduce participant or observer fatigue. During each break, observers will note down any thoughts regarding future questions or more in-depth observation. The pharmacists and technicians will be observed for 36 hours both before and after implementation, over a 72-hour period. We estimate observing tasks across 72 hours in each pharmacy [55], yielding 432 hours across the 6 participating pharmacies [57,60].

#### Data Analysis

To assess changes in pharmacists’ and technicians’ task and time distribution after implementation, we will calculate the proportion of total observed time in each task category by study period (before vs after implementation) for each pharmacy. The proportions of total observed time where pharmacists complete tasks with technicians or alone and use different information tools will also be calculated for each pharmacy. To assess the extent to which the introduction of MedSafeMap increased opportunities for pharmacists and technicians to engage with patients in CCM, we will examine changes in the time pharmacists and technicians spent in (1) having discussions with each other on responding to opportunities (ie, identifying patients with complex chronic health conditions who would benefit from CCM) and attending to unexpected threats (ie, personnel absences) and (2) having discussions with patients about medications [60]. *Z* tests for proportions will be used to compare the extent of before and after implementation change in the proportion of total observed time for each task category in each pharmacy site. A significance level of *P*<.05 will be used as well as the Holm approach to account for multiple comparisons, with 95% CIs based on the large sample normal approximation.

### Pharmacy-Reported Data

We anticipate that MedSafeMap will support pharmacists’ and technicians’ ability to embed CCM and other clinical services into their workflow, which will improve the quality of patient care, ideally, without increasing workload. To assess perceived changes in service provision, pharmacists and technicians will be administered a validated subjective workload survey [61]. Moreover, the number and type of documented medication-related problems during CCM services [62] that are identified and resolved for 6 months before and 12 months following MedSafeMap implementation will be collected. Pre-post comparisons of services provided and medication-related problems that they averted will be conducted.

### Pharmacist and Technician Evaluation

We also want to gain a more in-depth understanding of how MedSafeMap changed attitudes, behaviors, and performance. It is expected that pharmacists and technicians will feel more resilient after the implementation of MedSafeMap, that is, they will feel more “capable, facilitated, and supported by the organization to utilize resources to continually adapt and flourish at work, even if/when faced with challenging circumstances” [63]. This construct will be represented through the Employee Resilience Scale [63], a measure of behavioral elements, such as learning orientation, proactive posture, positive outlook, network leveraging, and adaptive capacity.

Pharmacists and technicians also are likely to eventually feel a greater sense of shared responsibility and teamwork, which would create an environment that enhances the medication safety of their patients. This construct will be represented through the National Aeronautics and Space Administration (NASA)–developed Cockpit Management Attitudes Questionnaire [64,65], comprising 3 scales, namely communication and coordination, command responsibility, and recognition of stressor effects, which have application for the pharmacy work system.

Because we are working with a small number of pharmacists and technicians and we want to conduct an in-depth exploration of pharmacy staff attitudes and behaviors, we will adapt the Employee Resilience and Cockpit Management Attitudes Questionnaire items into an interview guide that will include semistructured probes. In addition, 8 months following MedSafeMap’s implementation, interviews will be conducted using a retrospective pre- and posttest design approach [66,67] by asking, “Compared to before MedSafeMap was implemented...” This approach is appropriate because it is pragmatic and less time consuming, eliminates the impact of response-shift bias [66], and has been used successfully in pharmacy studies [68,69]. Questions will prompt insights into the impact of MedSafeMap on pharmacist and technician resilience, teamwork, and shared responsibility. Interview data will be subjected to the same rigorous qualitative data analysis techniques described in aim 1.

### Outcomes

Study outcomes are targeted to help populations of medication safety researchers and organizational leaders responsible for implementing proven practices. A summary of the anticipated outcomes can be found in Textbox 4.

**Anticipated outcomes.**

**Aim 1**

- Resilience narratives and functional resilience analysis method models that are context rich and context dependent
- Potential to uncover anticipated relationships between variables that can be tested in the subsequent aims to inform the development of the Medication Safety Map (MedSafeMap)

**Aim 2**

- Components of MedSafeMap that support chronic care management in community pharmacies
- MedSafeMap components that may include designs that realign currently existing tasks, coordinate tasks with different pharmacy staff members, or incorporate new practices
- MedSafeMap components that may include tools to facilitate pharmacist and patient and pharmacist and prescriber collaboration or to provide cognitive support for pharmacists and technicians to anticipate crises or other problems

**Aim 3**

- Quantitative and qualitative evaluation of the impact of MedSafeMap on community pharmacy processes and outcomes
- Toolkit to assess capacity and resilience
- Implementation guide

### Project Duration

The anticipated duration of the project is from September 30, 2023, to August 31, 2027. Activities concerning aim 1 were anticipated to end by August 31, 2024, but data collection has extended beyond that date. Activities concerning aim 2 were planned to commence on September 1, 2024, but began in February 2025 and will conclude in December 2025. Activities concerning aim 3 will begin on January 1, 2026, and last till August 31, 2027. Participant recruitment is expected to be completed by the conclusion of aim 3, alongside data collection. Results from varying analyses within the project are anticipated to be disseminated during the project’s duration and following its completion. Aims 1 and 2 have received IRB approval, and aim 3 will be reviewed by the University of Wisconsin-Madison IRB before recruitment begins.

## Results

As of August 2025, all 6 pharmacy sites have been recruited. In total, 3 of the 4 rounds of observations, 2 rounds of interviews with 12 pharmacists and 12 technicians from the study sites, and he 6 focus groups have been conducted. Preparations for the simulations are ongoing. Preparations for aim 3 will begin closer to November or December of 2025. Recruitment for aim 3 will begin once it has received approval from the IRB.

## Discussion

### Anticipated Findings

The prevailing safety-I approach for improving medication safety has emphasized retrospective incident analysis, reactive measures targeted at past problems, human error as an explanation, and control of work through procedural compliance and outcome monitoring [70]. This approach has proven to be insufficient and frustratingly slow at achieving zero harm in outpatient settings and specifically the community pharmacy [71]. Such a lack of success stems from investigating medication errors through root cause analysis, which assumes a shortsighted linear cause-and-effect model rather than a complex model [72]. Finally, importantly, these approaches create a negative psychological impact on staff by creating a culture of blame [31,73,74].

### Strengths

This study will be the first to operationalize resilience engineering to include a safety-II approach to medication safety in community pharmacies. Most of the safety studies involving community pharmacies assume a linear work process, which limits understanding of community pharmacies as complex work systems. Our research demonstrates that work is not conducted linearly but rather is composed of interdependent processes [75]. The use of the innovative FRAM model will provide a robust model to map multiple processes and their variability, which can inform co-design of potential solutions [36,76,77].

This project will prioritize and emphasize the role of pharmacy technicians as critical members of the community pharmacy work system. Technicians are increasingly being recognized for their willingness to assume new roles and adapt to changing environments [78] which was expedited during the COVID-19 pandemic. Technicians have proven themselves effective in collecting patient medication histories [79], performing administrative functions associated with vaccinations, and even providing immunizations to patients [80].

Pharmacist provider status, a new legal designation authorizing Medicaid reimbursement for pharmacists’ services, presents a timely opportunity to evaluate how increased or broadened responsibilities can be adopted while improving quality of care and medication safety. Legal endorsement to stretch professional boundaries and envision innovative designs [53] is the perfect circumstance to move the needle on outpatient medication safety through the design of the next generation of community pharmacies. Pharmacists and technicians, depleted by the unrelenting workload and additional responsibilities since the COVID-19 pandemic, find the safety-II approach to be attractive [71]. Safety-II also better represents and supports a culture of safety because it accurately portrays the constant variability of pharmacy work, the need for adjustment, and the importance of flexible adaptation in producing outcomes.

Introducing and using a safety-II approach will provide the test bed, tools, metrics, and frameworks to build the ENRICH PSLL. This new paradigm of approaching CCM with resilience engineering methodologies to improve quality in community pharmacies could then be disseminated to and adopted by medication safety researchers around the country.

### Limitations

Participant bias caused by sensitivity to the researcher’s presence is a recognized risk in qualitative observational studies, but it can be minimized by ensuring that observers have frequent presence in the research setting, which can lead to habituation to their presence and improvement in the researcher’s sensitivity to and respect for pharmacy staff’s concerns, and their ability to build relationships and trust. The risk of measurement bias will be reduced by having 2 researchers collect data only after achieving a satisfactory interrater reliability rate, ensuring that differences in interpretation can be identified, discussed, and resolved before full data collection is accomplished.

We will use 1 simulation site, despite being aware that community pharmacies have very different work systems. We also know that using multiple simulation sites would increase the generalizability of findings and enable comparison between sites. However, using simulations to evaluate prototypes that focus on workflow changes related to CCM is unprecedented in community pharmacies, so we want to begin with a single setting to ensure a robust infrastructure to evaluate such simulations. To expand our understanding beyond the single work system exemplified by the simulation site, we will seek input from advisory board members who will provide feedback on the feasibility of implementing MedSafeMap in their diverse community pharmacy settings.

### Conclusions

Community pharmacies have experienced a great deal of change in the last decade alone. With an increase in responsibilities due to the COVID-19 pandemic as well as changes to the health care structure that increase their workload, pharmacy staff are becoming increasingly burnt out. Coupled with high turnover rates and understaffing concerns, there is an unprecedented risk of safety errors and resulting patient harm. To allow the pharmacy staff to take on these responsibilities as CCM health care providers, we must find a way to manage the workload and optimize resources. We are in a unique position to provide support directly to the pharmacies and give them a voice to influence change in the pharmacy work system. Through this PSLL, we can rethink the current safety approaches and develop an improved work system for medication safety, resulting in improved work conditions for pharmacy staff and increased patient care.

## Appendix

Multimedia Appendix 1 Aim 1: pharmacy staff interview guide round 1.

Multimedia Appendix 2 Aim 1: pharmacy staff interview guide round 2.

## Abbreviations

ADE: adverse drug event
CARE: concepts for applying resilience engineering
CCM: chronic care management
ENRICH: Engineering Resilient Community Pharmacies
FRAM: functional resilience analysis method
IRB: institutional review board
MedSafeMap: Medication Safety Map
NASA: National Aeronautics and Space Administration
PSLL: patient safety learning laboratory
SEIPS: systems engineering initiative for patient safety
WAD: work as done
WAI: work as imagined
WOMBAT: work observation method by activity timing

## Data Availability

The datasets generated or analyzed during this study will be made available through the Interuniversity Consortium for Political and Social Research, a CoreTrustSeal certified repository, in adherence with the National Institutes of Health Data Management and Sharing policy.
