# Supplementary material for: Engineering Resilient Community Pharmacies for Chronic Care Management: Protocol for the Development of a Medication Safety Map
Source: JMIR Res Protoc. 2025 Sep 11;14:e69011. doi: 10.2196/69011 (PMC12464505; doi:10.2196/69011)
Supplement: Multimedia Appendix 1 [file resprot_v14i1e69011_app1.pdf]

ENRICH

Aim 1: Pharmacy Staff Interview Guide

INTRODUCTION
Thank you for agreeing to participate in this interview. Your experience and opinions are very important to us. Your participation will help us understand how community pharmacists and technicians do their work to keep their patients safe. Were you able to review the information sheet? (If not, verbally review the information sheet with participant at this time). As a reminder your participation is voluntary. This interview is being recorded so that we can fully capture your thoughts on this topic, but what you say is confidential and will not be shared with your employer under any circumstances. Your name and organization will not be identified in reporting the findings of this study. You can use examples when answering questions, but please do not include any patient names or identifying information. If you do accidently share any identifying information, we will make sure to redact it. The interview may take up to 45 minutes. Thank you for giving us this time. What questions do you have before we begin?

Part 1: Critical Incident Technique (20-25 minutes)

Adapted from "instance based" section of interview guide Hegde, S., Hettinger, A. Z., Fairbanks, R. J., Wreathall, J., Wears, R. L., & Bisantz, A. M. (2015). Knowledge Elicitation for Resilience Engineering in Health Care. *Proceedings of the Human Factors and Ergonomics Society Annual Meeting*, *59*(1), 175-179. https://doi.org/10.1177/1541931215591036

Health care is a complex and challenging field where patient safety is a major concern. Resilience in health care means overcoming the challenges and risks you encounter in everyday situations to ensure that the patient is safe. One of our goals with this project is to better understand how pharmacy staff anticipate or respond to situations in order to keep patients safe. We like you to help us understand, through an example from your own experience working in your current pharmacy where you or your team either:

- Handled a challenging or complex patient situation to help ensure the safest possible outcome
- Anticipated potential harm to a patient and took necessary steps to reduce that harm
- Noticed a discrepancy during a process and helped prevent an adverse event

1. Please describe an example of when this occurred including the background, sequence of events, people involved, and how the situation came under control.

Interviewer repeats back description of the case to (i) establish a coherent structure in terms of the various elements, sequences and processes involved (ii) confirm the details and build shared awareness of the case (iii) clarify inconsistencies, fill in 'gaps' or missing facts.

2. Next, I would like to ask you some additional questions about the example you provided:

MONITORING	
Problem Detection	How did you become aware that there may be a problem?
Checklist	Was there a checklist or written procedure used to recognize the event, was it from experience, or just something you realized in the moment while working with the issue?
Previous Experience	How did prior experience contribute to your assessment of the situation and your decision?
COMMUNICATION	

ENRICH

Communication	What kinds of communication took place between you and your colleague(s) and at what point?
RESPONDING	
Knowing what to do	What was it about the situation that let you know what to do? Was there a standard protocol specifically for such a situation?
Constraints	What were the main challenges or concerns and how were they overcome?
What Went Right	What is it that you and/or your team did right that helped bring the situation under control or prevent patient harm?
Resources	What resources (e.g. time, materials, technology, people, expertise, standards/protocols) were/are necessary or helpful in this situation?
Adaptive Measures	As the situation changed, how did you address it?
ANTICIPATING	
Expertise	How did your expertise help you to anticipate the problem?
Acceptability of Risk	What were the risks you identified? How did you weigh the risks and benefits of the action you took in this situation?
LEARNING	
Continuity	What is done to ensure that all staff members can deal with such a situation? [e.g. a sticky note, recorded documentation, staff meetings]

Thank you for sharing that example. Next, I'm going to ask you some more general questions.

Part II: General Questions

3. We know that many things can interrupt your workflow, and that pharmacy staff often have to change and prioritize tasks in response to real-time demands.
    a. How do you keep track of things when you decide to switch tasks?
    b. How do you keep track of things when you are interrupted by someone or something else?

4. How predictable is your work?
    a. What influences the predictability of your work?
    b. What do you do when something unexpected happens? [For example, a new urgent task, an unexpected change of conditions, a resource that is missing, something that goes wrong, etc.]

5. Things can happen that cause you to be unable to complete a prescription fill (examples: reimbursement/insurance problems, supply issues, partial fills, waiting for a prescriber to clarify questions, waiting on a confirming piece of information (date of birth, allergies, etc.)).
    a. What do you do when information is missing?
    b. What do you do when you cannot get ahold of certain people?

6. How are staff conflicts handled in the pharmacy?
    a. For example, when a difficult situation occurs when staff have different approaches to solving the problem?
    b. How do team members support each other?

ENRICH

7. What is one process or strategy your pharmacy uses that makes your job easier?

8. As we wrap up, do you have any other thoughts about the issues that we have discussed that you would like to share?
