# Supplementary material for: Engineering Resilient Community Pharmacies for Chronic Care Management: Protocol for the Development of a Medication Safety Map
Source: JMIR Res Protoc. 2025 Sep 11;14:e69011. doi: 10.2196/69011 (PMC12464505; doi:10.2196/69011)
Supplement: Multimedia Appendix 2 [file resprot_v14i1e69011_app2.pdf]

ENRICH

Aim 1: Pharmacy Staff Interview Guide Round 2

INTRODUCTION

Thank you for agreeing to participate in this interview. Your experience and opinions are very important to us. Your participation will help us understand how community pharmacists and technicians do their work to keep their patients safe. Were you able to review the information sheet? (If not, display the information sheet and review with participant at this time). As a reminder your participation is voluntary. This interview is being recorded so that we can fully capture your thoughts on this topic, but what you say is confidential and will not be shared with your employer under any circumstances. Your name and organization will not be identified in reporting the findings of this study. You can use examples when answering questions, but please do not include any patient names or identifying information. If you do accidently share any identifying information, we will make sure to redact it. The interview may take up to 45 minutes. Thank you for giving us this time. What questions do you have before we begin?

Non-Dispensing List

Working in a pharmacy involves much more than dispensing prescriptions. Pharmacies were recently granted provider status under Medicaid. With this status, pharmacies will be able to apply for remuneration for a number of services provided within the pharmacy. Below is a list of non-dispensing services compiled from our observations and the first round of interviews with staff from six different pharmacies. Please take a few minutes to review this list.

• **Vaccinations/Immunization and Non-Immunization Injections**<br><br>• **MedPack/Adherence Tools**<br>- MedSync<br>- MedPack scheduling (monthly/weekly)<br>- Routine MedPack Patient Check-ins<br><br>• **Durable Medical Equipment**<br>- Compression socks<br>- CPAP<br>- Shoe fitting<br>- Canes/walkers/crutches<br>- Wheelchairs<br>- Ostomy products<br>- Oxygen<br>- Bathroom aids for seniors<br><br>• **Screenings**<br>- Genetic Testing<br>- Colon Screening<br>- Strep screening<br>- Flu/RSV/covid screening<br>- Vitamin D screening<br><br>• **Connection to social services**<br>- Financial assistance programs<br>- Medicaid/Insurance assistance	• **Primary Care/Complex Care Management**<br>- *Diabetic Care*<br>o Lancet use<br>o Blood sugar meter<br>o Consult on lowering blood sugar<br>o Pump management<br>- *Respiratory Care*<br>o Asthma consult<br>o Nebulizer fitting<br>o Smoking cessation services<br>- *Cardiac Care*<br>o Blood pressure<br>o Blood thinning- INR Review<br>- *Pain Management*<br>o Opioid discontinuation assistance<br>o Naloxone<br>- *Weight Management*<br>o Weight loss programs<br>o Weight loss medications<br>- *Gender/Sex specific services*<br>o Reproductive considerations<br>o Women's Health<br>o LGBTQIA+ services<br>- *Medication Management*<br>o Confirming current medications<br>o Noncompliance reporting to physicians<br><br>• **Transitions of Care/Medication Reconciliation**<br>- Hospital to Home<br>- Hospice<br>- Hospital to long-term care

- Housing assistance<br>- Access to healthy foods<br>- Employment Assistance<br>- Mental health services referrals<br>- Substance use services referrals	

<u>Based on this list:</u>

1. What non-dispensing services are missing?

2. What have we included that shouldn't be there?

3. How do non-dispensing services impact your dispensing responsibilities?

4. Which non-dispensing services do you engage in the most frequently?

5. Which two of these activities is most difficult and why?
   a. What would make performing these activities easier?

6. Which two of these activities do you do now and you think you do well?
   a. What is your process now for doing [X]?
   b. What is your process now for doing [Y]?

7. Which two of these activities could be done better/improved?
   a. What is your process now for doing [X]?
   b. What is your process now for doing [Y]?

8. What is something you aren't doing currently, but is a need in your community?
   a. What are the barriers to providing this service?
   b. If those barriers could suddenly disappear, how would this service look in your pharmacy? Who would be involved? What types of resources would be needed?

9. Are you aware of any reimbursable non-dispensing activities that your pharmacy plans to implement in the future?

10. As we wrap up, do you have any other thoughts about the services that we have discussed that you would like to share?
